# Keeping a modeling-driven public health dashboard relevant—lessons learned from the California Communicable diseases Assessment Tool

**DOI:** 10.3389/fpubh.2025.1658645

**Published:** 2025-09-24

**Authors:** Mugdha Thakur, Lauren A. White, John Pugliese, David Crow, Phoebe Lu, Natalie Linton, Ryan McCorvie, Sindhu Ravuri, Hector M. Sánchez C., Brent Siegel, Jason Vargo, Tomás M. León

**Affiliations:** California Department of Public Health, Richmond, CA, United States

**Keywords:** infectious disease modeling, digital health, CalCAT, ensembling, California (USA), dashboard

## Abstract

Researchers rapidly developed modeling-based dashboards to support the global COVID-19 pandemic response, and this output has continued for other public health responses. These dashboards are often abandoned or deprecated over time, creating challenges for public health jurisdictions that would like to leverage them for decision-making. Maintaining a relevant and sustainable dashboard requires significant effort and attention to collating modeling results and meeting local public health needs. The California Communicable diseases Assessment Tool (CalCAT), a public-facing infectious disease modeling dashboard, demonstrates the key components to sustainability and relevance: robust and flexible workflows, cultivation of trust and user engagement, wide-ranging collaboration, and content reproducibility. The experience of CalCAT’s initiation and development highlights the need to be responsive to ever-changing stakeholder requests and to balance trade-offs in design choices for how modeling results are processed, presented, and shared.

## Background

As part of the COVID-19 pandemic response, many jurisdictions deployed dashboards displaying a variety of metrics including cases, hospitalizations, and vaccine uptake (1). Building upon these publicly available data sources and open repositories (2, 3), researchers rapidly developed mathematical models to estimate and forecast COVID-19 burden. These models varied by data source, time scale, geographic resolution, and target (e.g., cases, hospitalizations, deaths), requiring policymakers to contextualize and integrate predictions from multiple models. Moreover, different United States modeling hubs were managed by separate collaborative teams and while some coordination across hubs existed, there was not always agreement in timing, targets, or outputs of these efforts (4–6). Additionally, sub-state resolution was rarely available, partially due to suboptimal model performance at the county level and the resources needed for modeling over 3,000 U.S. counties (7, 8). Extracting local, jurisdiction-specific modeling predictions relevant for policy and decision-making remained challenging and time-consuming.

Having sub-state modeling predictions provides critical information because each county or region may exhibit specific epidemiological patterns not reflected in overall state trends. For example, California is the most populous state in the United States (~40 million people) with significant variation in population density and demographics. Motivated by these potential localized differences, the California COVID Assessment Tool (CalCAT; calcat.cdph.ca.gov) was launched in June 2020 by the California Department of Public Health (CDPH) Modeling Team along with internal and external collaborators to inform state and local public health COVID-19 response efforts. CalCAT brings together all publicly available California-specific modeling outputs, ensembles them, and interprets results for end users. Modeling products displayed on CalCAT include effective reproduction number (“R-effective”) nowcasts, forecasts, and scenario models for California and its constituent counties and regions. The CalCAT target audience includes California residents, public health staff and leadership, and researchers. CalCAT has since been renamed to the California Communicable diseases Assessment Tool to reflect broader disease coverage including influenza, added in fall of 2022, and combined respiratory burden (COVID-19 and influenza), added in winter of 2023.

CalCAT has had a direct impact on public health decision-making. As one of the very few dashboards to include county-and region-level model outcomes, CalCAT modeling for intensive care unit (ICU) capacity projections was used to support the regional stay-at-home order in California in winter 2020–2021. Synthesized model output from CalCAT was used in weekly situational updates to California state government leadership throughout the COVID-19 pandemic response. CalCAT results provided leading indicator information (e.g., R-effective) and burden projections that informed public health decision making. For example, CalCAT informed and was complementary to the Blueprint for a Safer Economy in 2020–2021, which identified risk tiers for counties that governed non-pharmaceutical interventions such as business capacity restrictions. In 2020, CalCAT had over 200,000 unique users across California and around the world.

While many data dashboards were effectively deployed throughout the COVID-19 pandemic (1), CalCAT is unique in that it: (1) synthesizes modeling results at the state and sub-state level; (2) combines modeling results for multiple respiratory pathogens (COVID-19 and influenza); and (3) integrates results across multiple analytic products (i.e., nowcasts, short-term forecasts, and long-term scenario models). While many of the national forecasting or scenario modeling hubs address one or two of these functions, they do not address all three (4–6). To our knowledge, CalCAT is the only dashboard deployed by a public health department during the pandemic that integrated forecasting and analytics with real-time data updates. CalCAT is also unique for its longevity; sustaining necessary data feeds and codebases is challenging and resource-intensive, and their neglect has rendered many data dashboards obsolete (7). This Viewpoint describes and highlights necessary steps and collaborations for developing and sustaining an infectious disease modeling dashboard that effectively supports public health decision-making. The following sections summarize the: (a) foundational components (i.e., content and collaborations) of CalCAT, (b) pillars for keeping CalCAT relevant, (c) ongoing challenges and lessons learned, and (d) overall conclusions.

## Foundations: content and collaborations

### Content: data input, pipelines, and output

Models on CalCAT use public and restricted access datasets including, but not limited to, cases, testing, wastewater surveillance, hospitalizations, and deaths. Many of these state and county level public datasets are available on the California Open Data Portal,^1^ which include data dictionaries and relevant context around data collection. Restricted datasets include linelist-level public health surveillance data reported to CDPH, which are not shared publicly for data privacy and confidentiality reasons. For example, state-level hospital admissions are publicly available from the National Hospital Safety Network dataset and are utilized as a target by U.S. forecasting and scenario modeling hubs (7, 9, 10). To CDPH, this dataset is available at the facility-level, and these counts are aggregated at the county and regional scales for use in modeling pipelines.

Depending on data availability and relevance to stakeholders, CalCAT’s content at any given time may include R-effective nowcast estimates; short-term (one-to four-week) forecasts of hospital admissions, hospital census, ICU census, cases, and deaths; and long-term scenario models based on “what-if” assumptions regarding future factors such as vaccination uptake and emergence of disease pathogen variants. With these metrics shown in the same place, CalCAT helps contextualize the different types of modeling evidence by bringing them together, providing extensive technical notes, and when appropriate including summary metrics and interpretation of model outputs (e.g., determining whether COVID-19 transmission is increasing, decreasing, or stable based on the summary of all available R-effective models).

Through an automated pipeline, ensemble values for metrics of public health relevance are calculated from all available models. The relevant reported data, model results and ensembles are displayed on the ‘shiny’ Posit package-based CalCAT dashboard (11). CalCAT’s outputs are downloadable directly from the CalCAT interface or from the archived version of model trajectories on the California Open Data Portal (12).

### Collaborations: relationships with model contributors

Modeling-based insights for public health emergencies are important and modeling capacity within public health agencies can be limited (13). The Team works with internal and external collaborators to provide county and region level metrics that may not otherwise be available; these partnerships have been central to CalCAT’s continued success. The models displayed on CalCAT come from external partners and contributors, coordinated hub efforts, and internally produced models maintained by the Team. As part of the COVID-19 response, the Team also worked closely with other internal CDPH teams to produce timely models that accounted for changes in testing practices, vaccination coverage, variants, and other time-varying aspects of the pandemic. For example, collaboration with influenza subject matter experts at CDPH guided influenza model development and maintenance across multiple seasons.

External partnerships have deepened over time through both ongoing informal and official collaboration mechanisms such as hub initiatives, formal grant funding, and the UC Health-CDPH COVID Modeling Consortium^2^ (14). This Consortium was a major effort by the University of California (UC) system and CDPH to improve evidence-based public health decision-making during the COVID-19 public health emergency and thereafter (15). Regular seminars and smaller group meetings with UC partners were helpful for sharing information about emerging trends that would affect model design (e.g., variants of concern, waning immunity). Such collaborations were particularly helpful for county-and regional-scale modeling because these targets were underrepresented or nonexistent in national modeling hubs.

The Team maintains open lines of communication with various forecasting and scenario efforts including CDC FluSight, Scenario Modeling Hubs, CDC Center for Forecasting and Outbreak Analytics (CFA), CDC InsightNet centers, and other modeling networks. This engagement has involved contributing California-specific modeling results, weighing in on submission requirements or scenario specifications and how these could be improved for public health impact, and sharing data and resources to support collaborative modeling efforts (9).

## Staying relevant

Building trust and two-way relationships with users have been key for keeping CalCAT relevant. Trust has been built through bidirectional communication with end users; ongoing quality control and validation of results displayed on CalCAT; striving for principles of openness and reproducibility in CalCAT development; and maintaining flexibility and relevance in the face of changing modeling inputs.

### Bidirectional communication with users

CalCAT is tailored to its users by engaging in an ongoing, bidirectional exchange of information with local health jurisdictions (LHJs) and researchers. Communication is encouraged through a dedicated email inbox linked on the dashboard. The Team also meets with LHJs during a biweekly “CalCAT Open House” meeting to discuss models, review other analytical products that may not be publicly available, exchange situational information, and receive feedback in real-time. Attendance has remained robust even after the end of the COVID-19 public health emergency, with more than one-third of California’s 61 LHJs regularly represented and more than one-half occasionally attending. These check-ins feature presentations on cutting-edge developments in infectious disease modeling methods, serving as a source of continuing professional development for participants. These meetings engage individual users and teams and provide an opportunity to conduct biannual surveys to inform future development goals. Some examples of regular survey questions are: “how often do you use the different CalCAT products (nowcasts, forecasts, scenarios) at the various geographical scales (state, region, county)?,” “which additional diseases are you interested in seeing on CalCAT?,” and “in what other areas would your jurisdiction benefit from modeling support?” Results from these dialogues have driven CalCAT modifications including expansion to new pathogens, aesthetic and accessibility choices for visual displays, and prioritization of model targets (e.g., hospital census vs. admissions). Infectious disease modeling results are additionally shared via academic literature (15–18) and in research collaborations, where they undergo scientific review but not user acceptability testing. Public health agencies are increasingly requesting user feedback for their digital products to ensure that messages are being received as intended and that websites are user-friendly and follow usability principles (19–21).

### Data processing, quality control, and validation

A daily, automated process checks for updated modeling results; these results are imported through a pipeline that includes scraping of public websites, downloading from GitHub and other repositories, and more secure data transfer such as through cloud object storage. Preliminary model results are then processed to generate ensembles and calculate uncertainty, and output is pushed to a development version of CalCAT. After review, the final production version of CalCAT is updated for public viewing. This multi-step process limits the publication of incorrect data and poorly calibrated models on CalCAT.

The Team conducts ongoing peer review and quality control of modeling results, as well as retrospective evaluation of model performance (17). The Team also receives inquiries from local public health officials, the media, and the public. Most of these communications cover data or modeling anomalies spotted on CalCAT and serve as an additional safeguard for CalCAT quality assurance and control. Other inquiries request more information or interpretation, which may be handled by the Modeling Team or redirected to the CDPH Communications Office.

### Adaptability and responsiveness

Several key structural features enable CalCAT to remain relevant, respond rapidly to changing input data streams and modeling outputs, and undergo routine updates for situational awareness. To maximize the use of all available model outputs for California, CalCAT is set up to process all file types and data types with no standardized template. Since many CalCAT inputs are generated independently by external collaborators, there is high variation in the cadence of updates and in the geographical coverage of available models. Moreover, the set of contributing models has varied substantially since CalCAT’s founding in 2020; different modelers stopped generating new model results or updated models in response to evolving disease and public health data landscapes. Backend data pipelines and workflows for CalCAT are designed specifically to be robust to these variations so that the most up-to-date information is available on the website.

### Sustainability and reproducibility

The Team aims for the contents of CalCAT to be FAIR—Findable, Accessible, Interoperable, Reusable (22). Extensive technical notes describe all constituent models with links to relevant underlying data, methodologies and code repositories. A notable success has been the willingness of the vast majority of external model contributors to publish their outputs publicly. Furthermore, the source code for a minimal reproducible version of CalCAT is maintained on GitHub for use by other teams and jurisdictions (23). This archiving is helpful for data and model versioning, and to support retrospective analyses (17). The biggest challenge in maintaining an archive of CalCAT products has been variability in formatting of the input and output datasets because of ongoing changes in data availability and stakeholder priorities. Anticipating and adapting to this variability in the data and modeling pipelines was an important lesson that emerged from CalCAT’s evolution.

Adherence to FAIR principles entails a long-term commitment to building trust between the maintainers and stakeholders and a shared vision of evidence-based preparedness and response activities for outbreaks. Sustainability of this effort relies heavily on ongoing prioritization by CDPH, and availability of personnel and funding. The early days of the Team relied on redirected, deployed, and volunteer staff. Initial funding came from the COVID-19 emergency response and was transitioned, when possible, to more permanent sources of public health funding as the team evolved over the following years. The size of the team has fluctuated from four to ten staff members, and over time more of them had formal training and a background in infectious disease modeling. The responsibilities of the team require both extensive technical knowledge and familiarity with the functions and operations of government public health within the capacity of available information technology (IT) resources.

## Challenges and lessons learned

The Team has learned valuable lessons throughout CalCAT’s development. First and foremost, flexibility to accommodate stakeholder requests for changing inputs over time is essential. For example, COVID-19 ICU capacity was initially more important as a forecasting target than inpatient capacity, but that reversed after treatment protocols improved the standard of care and ICU burden decreased. Similarly, case forecasts were deprioritized as testing practices changed and model performance declined. User surveys conducted with LHJs have shown that stakeholder priorities and data visualization preferences are not uniform, highlighting the importance of balancing limited staffing resources with stakeholder priorities.

The Team also learned that there is a tradeoff between complexity and clarity. This lesson was especially relevant when ensembling models to come up with a single best estimate for a given indicator. The team tried sophisticated model boosting techniques (machine learning, optimal weighting, etc.) for combining constituent models, but the median ensemble outperformed these more advanced methods in most cases; this is a consistent finding in the infectious disease modeling field (24, 25) and is standard practice for other forecasting hubs (7, 9, 26). Additionally, the median ensemble method is robust to the non-standardized reporting format of models submitted to CalCAT (e.g., including different quantiles for forecasts), which helps to ensure the consistency of the outputs. Parsimony has the added virtue of being easier to explain to non-technical audiences. Public health leadership has consistently preferred the clear messaging of ensemble models for sharing results with stakeholders, compared with showing multiple individual models or alternating between individual models.

Highlighting the uncertainty inherent in disease modeling and level-setting expectations was important for communicating with the general public. A September 2020 *Sacramento Bee* article addressed technical and communication challenges for projecting COVID-19 deaths in 2020 in smaller counties (27). Disease modeling—especially projecting trends out more than a few weeks, anticipating inflection points, estimating rates in less populous areas, accounting for uneven uptake of prophylactic interventions—is very difficult. Communicating this uncertainty visually (on the CalCAT dashboard) and verbally (in write-ups of CalCAT results, press releases, interviews, etc.) helps the public understand and be appropriately cautious when using CalCAT estimates.

One of CalCAT’s strengths is that it serves as a repository for all available models rather than relying on formatted submissions. However, this comes with challenges as staff are required to transform data, identify changing formats, and make decisions on whether to modify models to make them more comparable. Ensemble model interpretation can also be challenging when combining multiple models with heterogeneous methodologies. Additionally, a contributing model may suddenly stop updating, or an automated process may continue updating a model even if the results are clearly erroneous. Addressing these issues requires effort to monitor model outputs and contact model maintainers to assess these situations.

Alignment with other CDPH dashboards and reports is essential, so that end users do not get conflicting messages from CDPH about data trends. CalCAT relies on surveillance data but is not intended to be a primary means of reporting public health trends. However, it is often necessary to include observed surveillance data points to contextualize forecasting and scenario modeling results and to aid users in making their own interpretations about model calibration. The Modeling Team works with CDPH and LHJ collaborators to harmonize reporting across sources.

## Conclusion

While other dashboards sunsetted and disappeared, CalCAT has remained an active tool for infectious disease modeling and public health decision-making in California over the last 5 years, maintaining >99·9% uptime even as it has evolved. CalCAT is not as heavily used as it was during the early years of the pandemic, but it remains an important public-facing resource for situational awareness, analysis, and research, and its content can be easily pivoted in the event of a future public health emergency. CalCAT’s success and longevity are because it is more than just a dashboard; it is a commitment by CDPH to the provisioning and use of infectious disease models in California for public health practice and to the iterative process of constant improvement of said models and how they are shared and visualized. It is also a community of practice and collaboration between internal and external stakeholders committed to responding to the needs of local and state public health practitioners. CalCAT development priorities will continue to be driven by stakeholder and end user input. Five years into CalCAT’s existence, the dashboard has built on many lessons learned and will continue to improve to better serve the people of California through providing infectious disease modeling to support evidence-based public health practice.

## Data Availability

The original contributions presented in the study are included in the article/supplementary material, further inquiries can be directed to the corresponding author.

## Footnotes

^1^ http://data.ca.gov/

^2^ https://cpr3.ucsf.edu/modeling-consortium-meetings
